# Adoptive T Cell Therapy Is Complemented by Oncolytic Virotherapy with Fusogenic VSV-NDV in Combination Treatment of Murine Melanoma

**DOI:** 10.3390/cancers13051044

**Published:** 2021-03-02

**Authors:** Teresa Krabbe, Janina Marek, Tanja Groll, Katja Steiger, Roland M. Schmid, Angela M. Krackhardt, Jennifer Altomonte

**Affiliations:** 1 Klinik und Poliklinik für Innere Medizin II, Klinikum rechts der Isar, Technical University of Munich, 81675 Munich, Germany; teresa.krabbe@tum.de (T.K.); janina.marek@tum.de (J.M.); RolandM.Schmid@mri.tum.de (R.M.S.); 2 Institut für Pathologie, Klinikum rechts der Isar, Technical University, 81675 Munich, Germany; tanja.groll@tum.de (T.G.); katja.steiger@tum.de (K.S.); 3 Comparative Experimental Pathology, Klinikum rechts der Isar, Technische Universität München, 81675 Munich, Germany; 4 Klinik und Poliklinik für Innere Medizin III, Klinikum rechts der Isar, Technical University, 81675 Munich, Germany; angela.krackhardt@tum.de; 5 German Cancer Consortium of Translational Cancer Research (DKTK) and German Cancer Research Center (DKFZ), 69120 Heidelberg, Germany

**Keywords:** cancer, immunotherapy, oncolytic virus, fusogenic, adoptive T cells, melanoma

## Abstract

**Simple Summary:**

Cancer immunotherapy involves the application of strategies aimed at enhancing the body’s immune system to recognize and clear tumor cells. One such immunotherapeutic approach utilizes cytotoxic immune cells (T cells) harvested from the patient, which are expanded and activated, followed by re-infusion to the same patient. This process is termed adoptive cell therapy (ACT). Although effective in a limited setting, efforts are being made to improve these therapies through the development of rationally designed combination treatments. We have developed an approach, whereby tumors are pretreated with a virus, which has oncolytic effects on the tumor cells, in addition to modulating changes in the tumor microenvironment, thereby improving the recruitment of the adoptively transferred cytotoxic T cells and resulting in synergistic therapeutic responses in the tumor. This results in a substantial prolongation of survival, as demonstrated in an immune-competent mouse model of melanoma.

**Abstract:**

Cancer immunotherapies have made major advancements in recent years and are becoming the prevalent treatment options for numerous tumor entities. However, substantial response rates have only been observed in specific subsets of patients since pre-existing factors determine the susceptibility of a tumor to these therapies. The development of approaches that can actively induce an anti-tumor immune response, such as adoptive cell transfer and oncolytic virotherapy, have shown clinical success in the treatment of leukemia and melanoma, respectively. Based on the immune-stimulatory capacity of oncolytic VSV-NDV virotherapy, we envisioned a combination approach to synergize with adoptive T cell transfer, in order to enhance tumor cell killing. Using the immune-competent B16 melanoma model, we demonstrate that combination treatment has beneficial effects on the suppressive microenvironment through upregulation of MHC-I and maintaining low expression levels of PD-L1 on tumor cells. The approach led to additive cytotoxic effects and improved the recruitment of T cells to virus-infected tumor cells in vitro and in vivo. We observed substantial delays in tumor growth and evidence of abscopal effects, as well as prolongation of overall survival time when administered at clinically relevant dosing conditions. Our results indicate that treatment with oncolytic VSV-NDV, combined with adoptive T cell therapy, induces multi-mechanistic and synergistic tumor responses, which supports the further development of this promising translational approach.

## 1. Introduction

In recent years, major advances in our understanding of the tumor microenvironment and responses to therapy have led to a shift in the focus of cancer treatments from the reliance on monotherapies targeting tumor cells, to the development of rational combinatorial approaches involving new cancer immunotherapies to optimally support and unleash the endogenous anti-tumor immune response. Cancer immunotherapeutics involve numerous strategies to target different aspects of the tumor microenvironment and the innate and adaptive immune response to allow the tumor to be recognized and destroyed [1].

The successful induction of an anti-tumor immune response is illustrated in the cancer-immunity cycle introduced by Chen et al. in 2013 [2]. In short, tumor antigens released by dying tumor cells are taken up by antigen presenting cells (APCs), especially dendritic cells (DCs), that present these tumor antigens to naïve T cells in the lymph nodes, priming them for recognition and tumor cell elimination when they reach the tumor and infiltrate the lesion. If iterative expansion of the cancer immunity cycle is not inhibited, a broad anti-tumor immune response can achieve complete tumor clearance; however, multiple suppressive mechanisms, including masking of tumor antigens, infiltration barriers, and immunosuppressive signaling in the tumor microenvironment limit the ability of the anti-tumor immune response to effectively destroy the cancer [3].

Combination therapy can target multiple mechanisms, thereby supporting and stimulating the immune system on different levels. A rise in combinatorial clinical trials indicates the importance of these approaches in patient treatment [4]. Finding the right combination will depend on a profound understanding of the immune system, sophisticated bioinformatic analyses, and prediction systems as well as a multitude of well-characterized treatment options. Immunotherapeutic approaches, such as adoptive cell transfer (ACT) and oncolytic viruses (OVs) have been under preclinical development for years but have only recently proven their worth in the clinic. Chimeric antigen receptor (CAR) T cells targeting CD19 have been approved for therapy of leukemia [5], while an engineered herpes simplex virus expressing GM-CSF (T-Vec/Imlygic) has been approved for the treatment of late-stage malignant melanoma in 2015 [6]. Recently, their value in combination has also been under investigation, and evidence suggests the promising effects of oncolytic viruses in combination with the adoptive transfer of tumor-specific T cells, as summarized in detailed reviews [7,8,9]. In short, the multifaceted mechanism of oncolytic viruses enables debulking of the tumor, release of tumor-specific neoantigens, and an inflammatory change in the tumor microenvironment (TME). These factors improve the efficiency of ACT in solid tumors by promoting infiltration and prolongation of their cytotoxic activity with the aim of breaking the tumor’s immune tolerance and inducing a broad anti-tumor immune response, resulting in tumor clearance. Optimal success, however, is dependent on the selection of a safe virus platform that is both, highly oncolytic and immunogenic in its mediated cell death, as well as the design of an informed dosing schedule that leads to optimized synergistic responses.

We have recently reported that human CD8^+^ central memory T cells could be used as cell carriers to deliver oncolytic vesicular stomatitis virus (VSV) to human acute myeloid leukemia (AML) cells and exert cytotoxic effector functions to result in an effective combinatorial approach in an immune-deficient xenograft model [10]. Here, we focus on the potential of an enhanced and safer oncolytic virus platform in combination with adoptive T cell transfer in an immune-competent model as a further step towards clinical translation. The hybrid VSV-NDV platform was recently introduced by our group as an immune-stimulatory oncolytic virus, showing substantially enhanced safety and efficacy compared to VSV after systemic administration of an aggressive murine HCC model [11]. VSV-NDV was engineered by replacing the VSV glycoprotein within the VSV genome with the surface proteins of NDV to improve safety and introduce a fusogenic mechanism of action. In this construct, a polybasic cleavage site (F3aa) and the leucine to alanine substitution at amino acid 289 (L289A) lead to the hyperfusogenicity of VSV-NDV and efficient induction of immunogenic cell death [11]. The aspect of fusogenicity as a strategy to enhance oncolytic viruses as immunotherapeutics has recently gained attention [12,13]. Therefore, we hypothesized that the highly fusogenic capacity of VSV-NDV would make it an ideal candidate for the combination with other immunotherapeutic approaches, such as ACT.

In this study, we investigated the combination of oncolytic VSV-NDV with antigen-specific OTI T cells using the B16-OVA system, in which the mouse B16 melanoma cells express chicken ovalbumin as an immunogenic model antigen to facilitate immune monitoring. In vitro combination experiments revealed increased cytotoxicity in the tumor cells, compared to treatment with either monotherapy, as well as a specific homing of antigen-specific T cells to areas of virus replication. Furthermore, combination treatment led to the upregulation of MHC-I and downregulation of PD-L1 on the tumor cells, which indicates that the strategy could lead to enhanced antigen presentation and modulation of immune suppression, leading to additional immune-mediated effects. In vivo studies in immune-competent C57BL/6 mice bearing subcutaneous, syngeneic B16-OVA tumors demonstrated that VSV-NDV could mediate direct and abscopal tumor responses, but combination therapy with OTI T cells was the only effective approach when using a clinically relevant dosing scheme. The combination resulted in significantly delayed tumor growth and prolonged overall survival in this model. These results support the combination of oncolytic viruses with ACT as a rational combination, leading to multi-mechanistic and synergistic tumor responses for further development as cancer immunotherapeutics.

## 2. Materials and Methods

### 2.1. Cells and Virus

The murine melanoma cell line, B16, expressing the ovalbumin (B16-OVA) protein was cultured in DMEM GlutaMAX^TM^-I (Invitrogen, Carlsbad, CA, USA) supplemented with 10% heat inactivated fetal calf serum (FCS), MEM non-essential amino acids, sodium pyruvate, penicillin (50 U/mL) and streptomycin (50 μg/mL). The B16-F10 clone without the OVA protein was cultured analogously, and both B16 cell lines were kindly provided by Simon Heidegger (Klinik und Poliklinik für Innere Medizin III, Klinikum rechts der Isar, Munich, Germany). OTI T cells were isolated from the spleens of OTI mice (kindly provided by Melanie Kimm, Institute for Radiology, Klinikum rechts der Isar, Munich, Germany). The homozygous OTI mice produce T cells expressing transgenic Tcra-V2 and Tcrb-V5 T cell receptor chains in order to recognize the SIINFEKL peptide (ovalbumin residues 257–264) in the context of the H-2K^d^ MHC class I alloantigen. They were cultured and expanded in murine T cell medium (mTCM) consisting of RPMI GlutaMAX^TM^-I medium (Invitrogen, Carlsbad, CA, USA) supplemented with 10% heat inactivated FCS, MEM non-essential amino acids, sodium pyruvate, β-mercaptoethanol (50 mM) and activated with CD3/CD28 beads (Dynabeads, Thermo Fisher Scientific), as well as 40 U/mL IL-2, and 50 ng/mL IL-15. The green fluorescence protein (GFP) reporter gene was cloned as an additional transcription unit using unique restriction sites between the HN and L genes of VSV-NDV to generate the recombinant VSV-NDV-GFP construct. The virus was rescued and produced as previously described [11]. Virus stocks used for all experiments were produced in AGE1.CR.pIX cells (ProBioGen AG, Berlin, Germany) and purified by ultracentrifugation over a sucrose gradient. For animal experiments, sucrose was removed by ultracentrifugation and the concentrated virus was resuspended in PBS.

### 2.2. Growth Curves

The growth curve analysis of virus replication was determined in B16 cells. The cells were seeded at 70–80% confluency the day before infection. For infection, the cells were incubated with either rVSV-NDV-GFP at the indicated MOI or PBS as negative control for 1 h at 37 °C in PBS with Ca^2+^ and Mg^2+^. The cells were then washed 3x to remove remaining free virus particles, and the normal cell culture medium was added. A sample was immediately collected and used to determine baseline virus titers directly after infection and washing (0 h), and additional aliquots were collected at 16, 24, 48 and 72 h after infection for determination of virus titers via a 50% tissue culture infective dose (TCID50) assay in AGE1.CR.pIX cells.

### 2.3. Cytotoxicity Assay

Cytotoxicity was determined based on the release of lactate dehydrogenase (LDH) in cell culture supernatant. LDH was measured with the CytoTox 96 Non-Radioactive Cytotoxicity Assay (Promega, Madison, WI, USA), according to the manufacturer’s protocol. Absorption at 450 nm wavelength was detected with an absorbance microplate reader (Tecan, Austria). To obtain a maximum release control for each time-point, additional uninfected wells were treated with the supplied lysis buffer and incubated for 30 min, prior to collection of the supernatant. All experimental results were normalized to the maximum release control and performed in triplicate.

### 2.4. Microscopic Analysis

For visualization of the infection, representative images (bright field and fluorescence) were captured at 200× magnification using an Axiovert 40CFL microscope (Zeiss, Oberkochen, Germany) with an AxioCam ICm1 camera (Zeiss) attached to the microscope.

### 2.5. Co-Culture Experiments

The cells were pre-infected at the indicated MOI and incubated for up to 24 h, depending on the cell line susceptibility, before the addition of T cells. Supernatants were collected 16 or 24 h after the addition of OTI T cells to determine cytotoxicity via LDH analysis, as described previously. T cells and tumor cells harvested from these co-culture experiments were used to examine activation and tumor cell response by flow cytometry. For photomicroscopy of co-cultured cells, B16-OVA cells were seeded and infected with rVSV-NDV-GFP (MOI 0.1) for 16 h. T cells were labeled with CellTrace Far Red Cell Proliferation Kit (Thermo Fisher Scientific, Waltham, MA, USA) and added to the co-culture. Representative fluorescence images were captured after 4 h.

### 2.6. Flow Cytometry

Flow cytometric measurements were performed using the CytoFLEX S platform (Beckman Coulter, Brea, CA, USA). If not stated otherwise, samples were incubated for 25 min with FcR blocking reagent (Miltenyi Biotech, Bergisch Gladbach, Germany), washed in PBS, incubated for 1 h at room temperature with Viobility Fixable Dye, CD8, CD4 (Miltenyi Biotech, Bergisch Gladbach, Germany), PD1, IFNγ, and TNFα (BD Biosciences, Franklin Lakes, NJ, USA) staining antibodies or SIINFEKL(OVA)-/ (VSV-NP)-specific tetramers (MBL International, Woburn, MA), washed again and processed according to the manufacturers’ protocols. The compensation was performed based on staining results from UltraComp beads (Thermo Fisher Scientific, Waltham, MA, USA). The results were analyzed using Flow Jo (Ashland, OR, USA) software.

### 2.7. Animal Experiments

All animal experiments were performed in accordance with protocols approved by the institute’s Center for Preclinical Research and the regional government commission for animal protection (Regierung von Oberbayern, Munich, Germany). For survival analysis, six-week-old female C57BL/6J mice (Janvier Labs, Le Genest-Saint-Isle, France) were shaved and then injected with 2.4 × 10^5^ (right flank) and 1.2 × 10^5^ (left flank) B16-OVA cells subcutaneously in contralateral flanks. One week after tumor implantation (with an approximate tumor size of 20–50 mm^3^), the treatment was initiated. Intratumoral injections were only administered into the right tumor (injected tumor) in order to allow the observation of abscopal effects in the left tumor (uninjected tumor). The treatment schedules for the experiments are provided in the Results section or in the Supplemental Figures. Tumor width and length were measured regularly with a caliper, and the volume was calculated according to the modified ellipsoid formula: Tumor volume = 1/2(length × width^2^) [14]. Blood was collected on day 8 and 16 for flow cytometric analysis. The mice were monitored and euthanized at humane endpoints or at the latest when their tumor reached a diameter of 15 mm. Survival times with respect to the first injection of treatment were plotted in a Kaplan–Meier survival curve, and median survival times were calculated. Long-term surviving mice, together with age- and sex-matched control mice, were (re)implanted with 1.2 × 10^5^ B16-OVA cells to investigate memory immunity against the tumor.

For kinetics experiments, six-week-old female C57BL/6J mice were shaved and then injected with 2.4 × 10^5^ B16-OVA cells subcutaneously in both flanks. One week after tumor implantation, the treatment was initiated. The mice received virus (1 × 10^7^ TCID50) or PBS on indicated days and, depending on the treatment group, an OTI T cell injection (1 × 10^6^) by tail vein on day 1. Tumors and blood were harvested at specified time points after treatment. Tumors were divided in order to provide tissue for flow cytometry and immunohistochemistry (IHC). For flow cytometry, the tumor piece was mashed through a 40 µm filter after a 30-min incubation in Liberase TM (Roche, Basel, Switzerland) at a concentration of 20 µg/mL, and a single cell suspension was used for flow cytometry staining of infiltrating antigen-specific T cells. Alternatively, immune cells were concentrated via gradient centrifugation (20 min, 1025× *g*) in LymphSep (Biowest, Nuaillé, France) and used for flow cytometry staining. The measurement time was limited to 60 s in order to normalize each cell count to the size of the tumor before euthanization and correlate immune cell infiltration with tumor size. Blood was collected in EDTA-microvettes (Sarstedt Inc., Newton, NC, USA) and centrifuged (10 min, 1000× *g*). The resulting plasma was used to analyze the systemic cytokine profile after treatment with a bead-based cytokine array (Mouse Inflammation Panel, LEGENDplex, BioLegend, San Diege, CA, USA). Harvested spleens were mashed through a 40 µm filter and incubated with red blood cell lysis buffer for 2 min. After washing with mTCM and a second filtration step, the single cell suspension of splenocytes was frozen in 10% DMSO in FCS at −80 °C and thawn for the peptide activation assay.

### 2.8. Peptide Activation Assay

Frozen splenocytes collected from treated tumor-bearing mice at day 2, 5, and 8 after the first treatment were thawn and incubated in mTCM for 5 h at 37 °C. For the peptide activation assay, 2 × 10^6^ splenocytes were added to a 96-well plate prepared with peptide solution at a final concentration of 1 μg/mL OVA or TRP2 peptide. Brefeldin A was added to each well after a 1-h stimulation. After incubation for 15 h at 37 °C, the cells were transferred to a V-bottom 96-well plate and stained for extracellular CD4 and CD8, as well as intracellular IFNγ and TNFα after fixation and washing using BD Cytox and Perm/Wash Buffer. Staining was analyzed by flow cytometry.

### 2.9. Histology and Immunohistochemistry

For immunohistochemistry (IHC), tumor pieces were fixed in 4% paraformaldehyde overnight, followed by dehydration and embedding in paraffin. Then, 2 µm-thin slices were subjected to immunohistochemical staining using a rabbit monoclonal antibody against CD3 (DCS, Hamburg, Germany) or CD8 (Dianova, Hamburg, Germany) on a Bond RX automated staining instrument (Leica, Biosystems, Nußloch, Germany). An analysis of pathological changes and confirmation of positive immunohistochemical reaction were performed by a certified pathologist who was blinded to the treatment groups of the specimens.

### 2.10. Statistical Analysis

The data were plotted and analyzed using GraphPad Prism 7.0 (GraphPad Software, San Diego, CA, USA). Means and standard error of the mean (SEM) were plotted when applicable. Individual data points were compared for statistical significance using an unpaired Student’s t-test, and p-values of less than 0.05 were considered to be statistically significant (* *p* < 0.05, ** *p* < 0.005, *** *p* < 0.001, **** *p* < 0.0001). Survival data was plotted in Kaplan–Meier curves, and statistical significance calculated by log-rank test.

## 3. Results

### 3.1. B16-OVA Cells Are Susceptible to VSV-NDV Virotherapy and Targeted T Cell (OTI) Treatment

**Figure 1:**
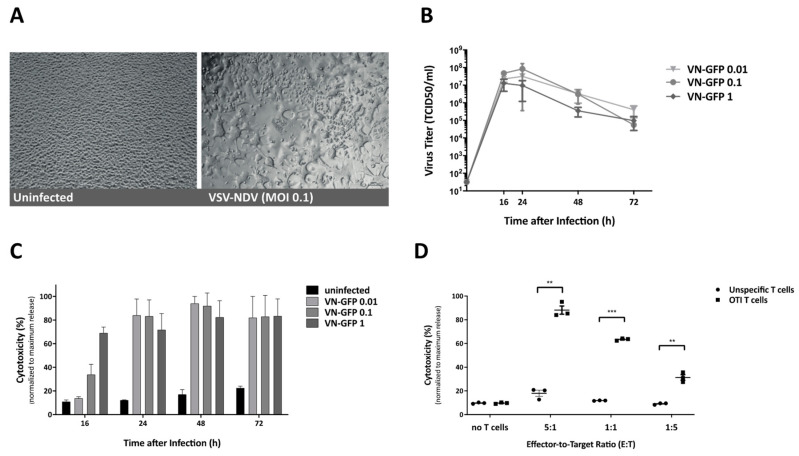
Infection of B16-OVA cells by virotherapy (VSV-NDV) and targeted cytotoxicity by T cells (OTI). (**A**) B16-OVA cells were infected with VSV-NDV at MOI 0.1 (right panel) or left uninfected (left panel), and images were captured 16-h post-infection. Representative images demonstrating characteristic syncytia formation in the infected well and the healthy uninfected monolayer were captured at 200× magnification. (**B**) B16-OVA cells were seeded and infected with VSV-NDV (VN) at different MOIs. Virus titers were determined via a TCID50 assay from tissue culture supernatants collected at indicated times after infection. (**C**) Corresponding cytotoxicity analysis was determined via an LDH detection assay and normalized to a maximum release control. (**D**) The cytotoxic effector function of OTI T cells on B16-OVA melanoma cells was determined by LDH assay. Cells were co-cultured with OTI or unspecific control T cells for 24 h in the indicated effector-to-target ratios. T cells were isolated from the spleens of OTI or C57Bl6 mice and expanded in vitro before addition to the co-culture. Additional wells of B16-OVA cells were left without the addition of T cells, in order to demonstrate the baseline level of cytotoxicity. All data are presented as mean + SEM of triplicate experiments, and statistical significance was determined by student’s t-test (** *p* < 0.01, *** *p* < 0.001).

In the first step, the susceptibility of the B16-OVA cell line to VSV-NDV infection and elimination by OTI T cells were examined separately. Growth curve analysis was performed to characterize viral replication in the B16-OVA cell line. Representative images were captured at 16 h after infection to visualize the fusogenic effect of rVSV-NDV-GFP. While control, PBS-treated, cells appeared as a healthy and confluent monolayer, cells infected with rVSV-NDV-GFP displayed areas of obvious syncytial formation (Figure 1A). Supernatant samples collected at various time-points post-infection were used to determine the amount of released infectious virus particles over time via TCID50 assay (Figure 1B). Maximum virus titers of up to approximately 10^8^ TCID50/mL were reached between 16- and 24-h post-infection, irrespective of the MOI used for the infection, indicating that the cell line was highly susceptible to infection with this virus. Virus titer decline at later time-points indicates a depletion of host tumor cells supporting virus replication. This was confirmed by subjecting the same supernatants to cytotoxicity analysis via an LDH detection assay (Figure 1C). Dose-dependent cytotoxicity in response to VSV-NDV infection was observed at 16 h post infection, while nearly complete cytotoxic effects were determined at subsequent time-points, regardless of the MOI applied. In a similar approach, the cytotoxic potential of OTI T cells on B16-OVA tumor cells was determined via LDH assay. Here, OVA-specific OTI T cells isolated from OTI mice were co-cultured with B16-OVA target cells in different effector-to-target ratios. Unspecific T cells isolated from C57BL/6 control mice were used as negative controls, and samples for LDH assay were taken 16 h after T cell addition to the co-culture. As expected, rapid and dose-responsive cell killing by OTI T cells was observed and confirmed by the LDH assay (Figure 1D). Based on these data, B16-OVA cells are very susceptible to killing by both oncolytic VSV-NDV and OTI T cells in vitro, leaving a small window for improvement by combination treatment.

### 3.2. Cytotoxic Effects of VSV-NDV and OTI T Cells Are Increased When Applied in Combination

**Figure 2:**
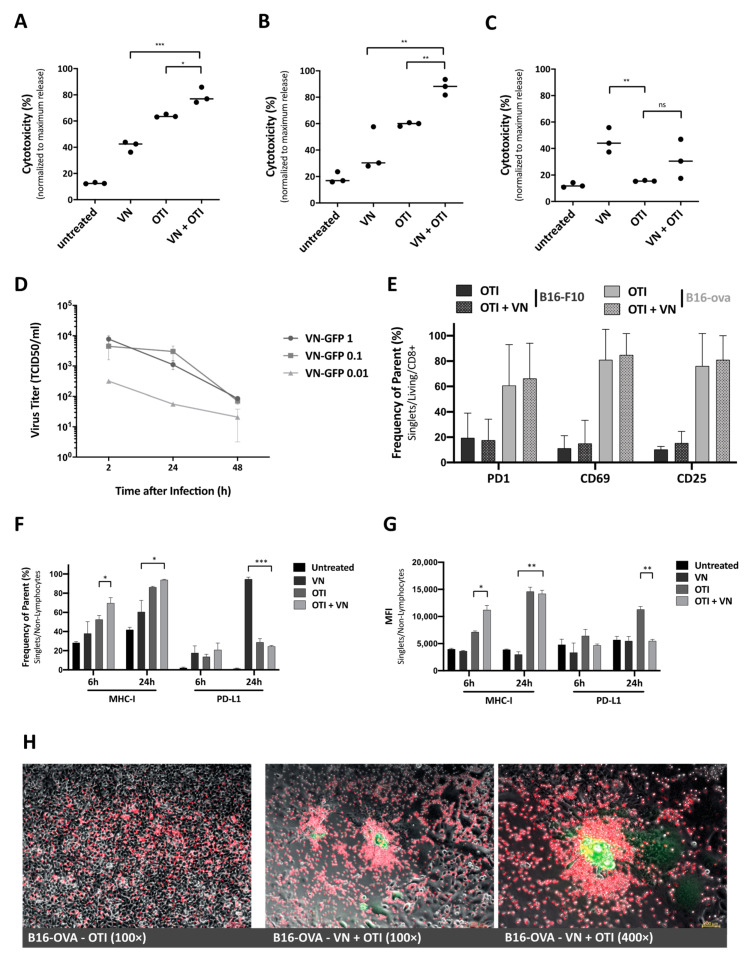
In vitro effects of VSV-NDV and OTI T cell combination therapy. Co-culture experiments were performed by seeding B16 cells and pre-infecting them with VSV-NDV (VN) at MOI 0.1 for 8 h, before adding OTI T cells in a 1:1 effector-to-target ratio. Supernatant samples were collected (**A**) at 8 h after OTI addition to B16-OVA cells or (**B**) 16 h after OTI addition to a 1:1 mixed B16 melanoma (B16-OVA: B16-F10) cell population to determine cytotoxicity by LDH assay. (**C**) Additional co-culture experiments were performed using B16-F10 cells pre-infected with VSV-NDV for 24 h prior to addition of OTI cells and collecting supernatant for LDH assay 16 h later. (**D**) OTI T cells were infected with VSV-NDV at the indicated MOIs, and supernatant samples were collected 2, 24 and 48 h after infection for virus titer determination by TCID50. (**E**) OTI T cell activation in response to co-culture with/without VSV-NDV pre-infection in B16-OVA and B16-F10 cells was determined by measuring surface expression of PD1, CD69, and CD25 via flow cytometry. Co-culture experiments were performed by seeding B16 cells, pre-infecting them with VSV-NDV (VN) at MOI 0.01 for 16 h before adding OTI T cells (OTI) in a 1:1 effector-to-target ratio. Cells were harvested 24 h after T cell addition and stained for flow cytometric analysis, or (**F**) tumor cells were harvested 6 and 24 h after OTI addition and stained for flow cytometric analysis of MHC-I and PD-L1 expression. (**G**) Mean fluorescence intensity (MFI) was determined from the same samples. All data are presented as mean + SEM of triplicate experiments, and statistical significance was determined by student’s *t*-test (* *p* < 0.05, ** *p* < 0.01, *** *p* < 0.001). (**H**) Labeled OTI T cells were used in co-culture experiments to visualize accumulation around VSV-NDV-induced syncytia. B16-OVA cells were treated with PBS (left panel) or infected with VSV-NDV-GFP at MOI 0.01 for 16 h (middle and right panel), and OTI T cells labelled with FarRed CytoTrace (ThermoFischer) were added. Representative pictures were taken 4–6 h later under 100× (left and middle) or 400× (right) magnification. Composites were generated using ImageJ.

To examine the potential of combination therapy in vitro, cytotoxicity was measured via LDH assay from supernatants from cells subjected to virus infection and T cell co-culture, in comparison to treatment with either monotherapy. B16 cells were seeded one day prior to infection, then infected at MOI 0.1 or left untreated. After 16 h, OTI T cells were added to a subset of wells in a 1:1 effector-to-target ratio, that was chosen to provide some room for improvement by the combination. Supernatant samples were analyzed for LDH after an 8-h co-culture. Comparing treatment groups revealed a significant increase in cytotoxicity after combination treatment, compared to either VSV-NDV infection or T cell co-culture alone (Figure 2A). This effect could be emphasized in a similar experiment using mixed B16 cells (B16-OVA and B16-F10 in a 1:1 mix). Here, we could observe a more substantial increase in cytotoxicity after a 16-h co-culture in the combination treatment, since the B16-F10 cells are not recognized by OTI T cells and are, therefore, only susceptible to the virus infection (Figure 2B). This setup mimics the heterogeneous tumor cell population that would be encountered in a clinical tumor setting. As a negative control, B16-F10 cells alone were used and treated as described above. To account for differences in the B16-F10 cell line susceptibility to VSV-NDV infection (Figure S1), the time of pre-infection was prolonged to 24 h. As expected, OTI T cells did not have an effect on B16-F10 cell viability, since these cells do not express the OVA peptide; however, VSV-NDV resulted in a significant cytotoxic effect (Figure 2C).

### 3.3. VSV-NDV Does Not Replicate in OTI T Cells or Change Their Activation Pattern

To determine whether VSV-NDV could inadvertently alter OTI T cell function, their susceptibility to infection and the expression of activation markers were examined more closely. TCID50 analysis from infected OTI T cells revealed a rapid reduction of virus titer in the supernatant, indicating that there is no replicative potential of the virus in these cells and confirming the tumor specificity of VSV-NDV (Figure 2D). Similarly, we have not observed murine T cell survival to be affected by the presence of VSV-NDV, indicating that LDH data obtained in co-culture experiments stems from target cell death. Furthermore, OTI T cells collected from co-culture experiments were analyzed for PD1, CD69 and CD25 activation marker expression by flow cytometry. No differences in T cell activation markers could be observed in response to VSV-NDV pre-infection, indicating that virus had no impact on T cell activation induced by tumor cell recognition (Figure 2E). As a control, B16-F10 cells subjected to the same experimental conditions, and co-cultured with OTI T cells, resulted in constitutively low levels of activation markers, either in the presence or absence of the virus, indicating that VSV-NDV does not cause non-specific activation of OTI T cells (Figure 2E).

### 3.4. Combination Treatment Enhances MHC-I Expression, While Maintaining Low PD-L1 Expression, on Tumor Cells

Despite its immune stimulatory potential, inflammation has also been shown to modify the tumor microenvironment by promoting immunosuppressive pathways, such as PD-L1 expression or MHC down-regulation through cytokine secretion. The effects of PBS, VSV-NDV, OTI, and VSV-NDV in combination with OTI treatment on these pathways were observed by measuring MHC-I and PD-L1 expression via flow cytometry. Tumor cells were harvested after 6 and 24 h of infection and/or co-culture, in the set-up described earlier. Combination treatment led to early upregulation of MHC-I expression and resulted in almost 100% MHC-I-expressing tumor cells (Figure 2F). Similarly, MHC expression levels on individual cells increased, as represented by mean fluorescence intensity (MFI) data (Figure 2G). The percentage of PD-L1 expressing cells was significantly increased shortly after VSV-NDV infection, but not in the combination setting with OTI T cells (Figure 2F). Tumor cells co-cultured with OTI and pre-infected with VSV-NDV seem to maintain low PD-L1 expression, possibly due to a reduced virus replication in the presence of activated T cells, as shown previously [10]. Low PD-L1 expression would indicate that this treatment strategy could help to avoid or delay T cell exhaustion [15]. A similar effect can be observed when looking at the mean fluorescence intensity (Figure 2G), indicating that not only overall expression, but also the expression level on individual cells remains low after the combination treatment (Figure S2). Differences in MHC-I and PD-L1 responses to treatment suggest the presence of independent activation pathways. Although both markers are reportedly induced by IFNγ, PD-L1 is also regulated by other cytokines/chemokines [15]. Taken together, the increase in MHC-I expression and maintenance of low levels of PD-L1 on tumor cells in response to combined VSV-NDV and OTI T cell therapy contribute to the potential synergy of the approach.

### 3.5. OTI T Cells Are Recruited to Sites of VSV-NDV-Induced Syncytia Formation

Microscopic analysis of the co-culture of OTI T cells with B16-OVA cells pre-infected with VSV-NDV revealed an accumulation of OTI T cells at sites of syncytia formation. To better illustrate these observations, T cells were fluorescently labelled for visualization in co-culture experiments. The accumulation of T cells around sites of rVSV-NDV-GFP infection could already be observed after 4 h (Figure 2H). In contrast, control wells with uninfected B16-OVA cells showed an even distribution of OTI T cells on top of the monolayer, with no apparent areas of preferential targeting (Figure 2H, left panel). Localization around syncytia indicates a specific recruitment of OTI T cells to infected cells, which we speculated could be induced by chemokine release from the tumor cells in response to infection; however, a chemokine analysis of B16-OVA cells infected with rVSV-NDV-GFP using an ELISArray did not reveal any significant differences in those chemokines analysed, compared to uninfected cells (data not shown), suggesting a different mechanism of recruitment. Nevertheless, an enhanced recruitment of T cells in response to VSV-NDV infection of tumor cells further supports the rationale for the enhancement of adoptive T cell therapy via pretreatment with oncolytic virus.

### 3.6. VSV-NDV Treatment Enhances the Anti-Tumor Effect and Complements ACT In Vivo

**Figure 3:**
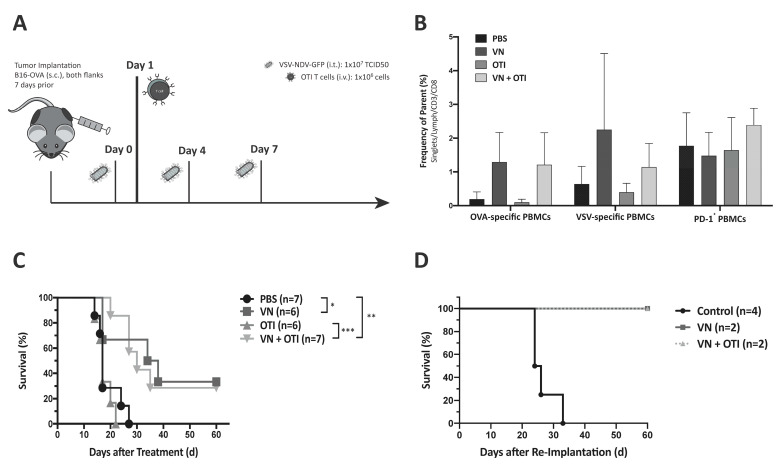
VSV-NDV and combination therapy improve survival in murine melanoma model. (**A**) Experimental set-up of the survival experiment. C57BL6/J mice were implanted with 2.4 × 10^5^ (injected tumor) and 1.2 × 10^5^ (uninjected tumor) B16-OVA cells subcutaneously on contralateral flanks. One week later, the mice were randomly distributed into treatment groups (*n* = 6–7) and injected intratumorally with VSV-NDV (VN) at a dose of 10^7^ TCID50 or PBS in an equal volume of 50 μL on day 0, followed by an intravenous OTI T cell injection (1 × 10^6^) on day 1 for combination treatment or OTI monotherapy. Intratumoral virus or PBS injections were repeated on day 4 and 7. (**B**) Blood was collected on day 16 after the first treatment to analyze the systemic expansion of OVA- and VSV-specific CD8^+^ T cells via tetramer staining as well as PD1 expression patterns by flow cytometry. (**C**) Survival of the different treatment groups was monitored and plotted in a Kaplan–Meyer curve. Statistical significance was determined by log-rank test (* *p* < 0.05, ** *p* < 0.01, *** *p* < 0.001). (**D**) Long-term surviving mice treated with VSV-NDV or combination therapy (VN + OTI) (N = 2) were subjected to rechallenge with subcutaneous implantation of 1.2 × 10^5^ B16-OVA cells in a 100 µl volume. Previously untreated age- and sex-matched C57BL6/J mice were similarly implanted with B16-OVA cells as a control. Survival was monitored and plotted in a Kaplan–Meier curve.

Based on the promising in vitro results, the combination therapy approach was then analyzed in vivo for the treatment of subcutaneous murine melanoma. The experimental set-up for survival analysis is illustrated in Figure 3A. To examine potential differences in circulating immune cells in response to the treatments, blood was collected on day 16 after initiation of treatment. Flow cytometric analysis of PBMCs confirmed that both VSV-NDV treatment alone, as well as in combination with OTI, tend to induce the presence of OVA-specific CD8^+^ T cells in the periphery, compared with PBS treatment (Figure 3B, left). An increase in VSV-specific CD8^+^ T cells could also be observed in both treatment groups receiving VSV-NDV, although there is a possible (non-significant) decrease in the combination group (Figure 3B, middle). PD-1 expression on peripheral CD3^+^ lymphocytes appear to be unaffected, regardless of treatment (Figure 3B, right); however, the PD-1 expression on tumor infiltrating lymphocytes is likely to be different than what is observed on PBMCs. Tumor growth was monitored regularly, and animals were sacrificed as soon as one tumor reached 15 mm in diameter. VSV-NDV, both alone and in combination with OTI T cells, resulted in significant survival prolongation compared to PBS or OTI monotherapy (Figure 3C). To evaluate the long-term protection of the anti-tumor immune response induced by combination therapy, surviving mice were subjected to re-challenge with B16-OVA cells, implanted subcutaneously. As a control, the same tumor cells were similarly implanted in naïve age- and sex-matched control mice. While the naïve mice quickly succumbed to tumor growth as expected, the survivors remained tumor-free and showed no sign of tumor growth over the duration of the experiment (Figure 3D), indicating the formation of a memory response. However, it should be noted that there were only 2 long-term surviving mice in each treatment group that could be used in the re-challenge experiment, and there were no observable differences between the tumor rejection in those mice that had previously received VSV-NDV alone compared to those receiving combination therapy, indicating that both groups seemed to be equally protected from tumor re-challenge in this experiment.

### 3.7. Combination Treatment Enhances Tumor Infiltration of CD8^+^ T Cells

To analyze the kinetics and mechanism of the treatment responses, mice were implanted with B16-OVA tumors on both flanks as in the previous experiment. One week later, VSV-NDV or PBS was administered intratumorally on the right side on day 0, followed by intravenous administration of OTI T cells or PBS on day 1. The mice were randomized for euthanasia on days 2, 5 and 8 for analysis of tumors, blood, and spleens (Figure S3).

**Figure 4:**
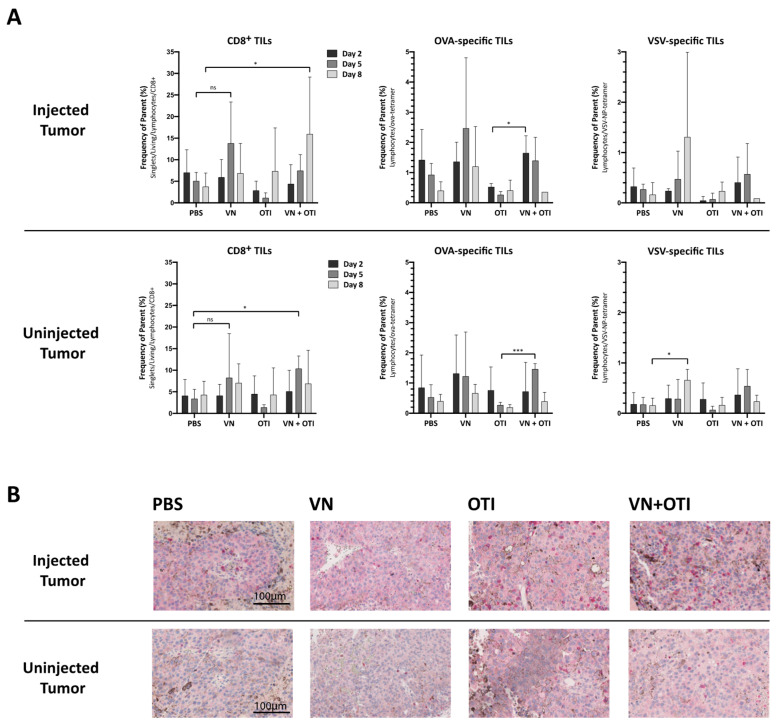
Features of tumor infiltrating lymphocytes in the injected and uninjected tumor. (**A**) Mice were subcutaneously implanted with 2.4 × 10^5^ B16-OVA cells on both flanks. One week later, the mice were randomly distributed into indicated treatment groups (*n* = 3–6) and injected intratumorally with VSV-NDV (VN) at a dose of 10^7^ TCID50 or PBS in an equal volume of 50 µL on day 0, followed by an intravenous OTI T cell injection (5.5 × 10^6^) on day 1 for combination treatment or OTI monotherapy. Tumors from both flanks were harvested on day 2, 5 and 8 after the first treatment. Single cell suspensions were generated from tumor tissues, and tumor infiltrating lymphocytes (TILs) were analyzed for CD8 surface expression and OVA- or VSV-specificity via flow cytometry. Data are presented as mean + SD, and statistical significance was determined by student’s *t*-test (* *p* < 0.05, *** *p* < 0.001). (**B**) Paraffin-embedded tumor sections of 2 µm in thickness were subjected to immunohistochemical analysis using antibodies specific for mouse CD8 and visualized using a fast red chromogen for detection. Representative sections of injected (top panel) or uninjected (bottom panel) tumors from the indicated treatment groups were captured at 200× magnification. Scale bars indicate 100 µm.

Early timepoints and a single virus injection were chosen to follow the effects of an intratumoral virus injection on the immune response, as well as the immediate effects on the adoptively transferred T cell population without the added complexity of multiple virus injections later. Tumor infiltration by CD8^+^ T cells is an indicator of immunogenicity and was correlated with slower disease progression and improved treatment outcome [16,17]. Especially in the injected tumor, an increase of CD8^+^ T cell infiltration in mice in the combination treatment group, compared to PBS treatment, was observed by day 8 by FACS analysis (Figure 4A, upper left panel). This trend is emphasized in immunohistochemical staining of CD8^+^ lymphocytes within tumor tissue isolated on day 8 after treatment from these mice (Figure 4B). Moreover, significantly enhanced infiltration of OVA-specific T cells was observed in both the injected and uninjected tumors after combination treatment compared to OTI monotherapy on day 2 and 5, respectively, by tetramer staining (Figure 4A, middle panel). The systemic trend observed previously was confirmed in the tumor, in that VSV-specific TILs were induced only by monotherapy with VSV-NDV in both the injected and uninjected tumors on day 8, and not by combination treatment (Figure 4A, right panel). With immunotherapy in mind, the systemic treatment response is an important indicator for immune-related adverse events associated with immunotherapies, especially cytokine release syndrome [18]. Only minor changes in cytokine concentrations in serum from these animals could be detected (Figure S4). The results of this experiment indicate that VSV-NDV induces both virus-specific, as well as tumor-specific immune reactions, and that abscopal effects could be driven by a virus-mediated systemic anti-tumor immune response, a concept which is supported by previous reports [19].

### 3.8. Survival Benefit of Combination Therapy Is Highlighted in a Clinically Relevant Dosing Schedule

**Figure 5:**
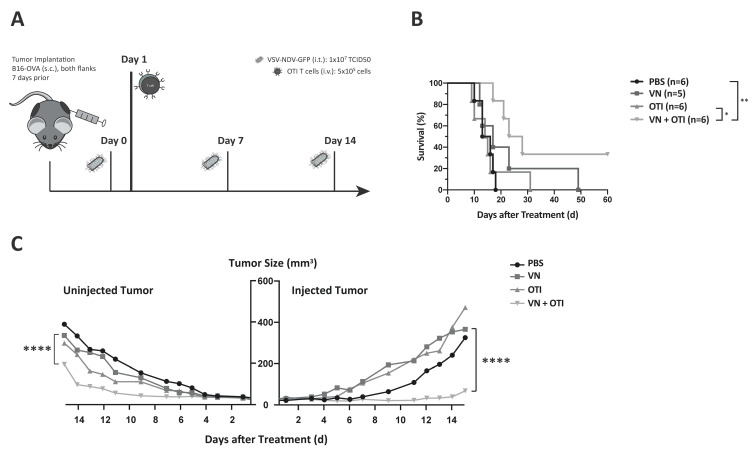
Benefits of combination therapy are highlighted in a modified dosing scheme. (**A**) Experimental set-up of the adapted survival experiment. C57BL6/J mice were implanted with 2.4 × 10^5^ (injected tumor) and 1.2 × 10^5^ (uninjected tumor) B16-OVA cells subcutaneously on contralateral flanks. One week later, mice were randomly distributed into treatment groups (*n* = 5–6) and injected intratumorally with VSV-NDV at a dose of 10^7^ TCID50 or PBS in an equal volume of 50 μL on day 0, followed by an intravenous OTI T cell injection (5.5 × 10^6^) on day 1 for combination treatment or OTI monotherapy. Intratumoral virus or PBS injections were repeated on day 7 and 14. (**B**) Survival of the different treatment groups was monitored and plotted in a Kaplan–Meier curve. Statistical significance was determined by log-rank test (* *p* < 0.05, ** *p* < 0.01). (**C**) Tumor growth was monitored by caliper measurements of tumor width and length. The volume was calculated according to the modified ellipsoid formula: Tumor volume = 1/2(length × width^2^). Mean tumor volume was plotted up to day 14 for uninjected and injected tumors according to the indicated treatment groups. Statistical significance was determined by student’s t-test (**** *p* < 0.0001).

A weakness of many preclinical studies using OVs is that they rely on frequent injections in order to achieve statistically significant effects, even though these dosing schemes are not feasible in clinical practice. This is an important contributing factor to the lack of translation of preclinical findings to clinical survival prolongations [20]. In order to mimic a more realistic dosing scheme for a clinical trial protocol, we adapted the virus dosing to weekly injections (Figure 5A), as opposed to applying 3 injections within one week. As expected, results from the survival analysis revealed a less effective VSV-NDV monotherapy, while the combination treatment resulted in a stable effect. Although the difference in median survival time was not statistically significant compared to VSV-NDV monotherapy, it is the only treatment group that resulted in a statistically significant survival prolongation compared to PBS. The fact that neither monotherapy resulted in a significant survival benefit under these conditions, indicates that the combination approach results in synergistic therapeutic effects. Furthermore, 2 mice receiving combination therapy achieved complete tumor clearance, despite a slight decrease in median survival time as compared to that achieved in the previous dosing schedule (25.5 days (Figure 5B) compared to 30 days (Figure 3B)). The survival benefit of the combination therapy is due to a significant delay in tumor growth in both the injected and uninjected tumor up to day 15 (Figure 5C). This difference is emphasized in the comparison of the individual tumor growth kinetics over time (Figure S5) and would suggest the involvement of synergistic mechanisms of tumor clearance in the VSV-NDV and adoptive T cell combination approach.

### 3.9. Enhanced Infiltration of Antigen-Specific T Cells after VSV-NDV Therapy Contributes to Tumor Cell Clearance in a Clinically Relevant Dosing Scheme

**Figure 6:**
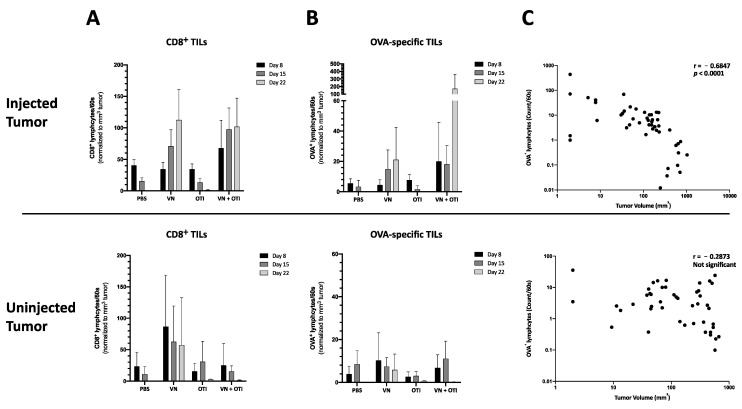
Treatment effects of tumor infiltrating lymphocytes in the injected and uninjected tumor. Mice were subcutaneously implanted with 2.4 × 10^5^ (injected tumor) and 1.2 × 10^5^ (uninjected tumor) B16-OVA cells subcutaneously on contralateral flanks. One week later, mice were randomly distributed into indicated treatment groups (*n* = 5) and injected intratumorally with VSV-NDV (VN) at a dose of 10^7^ TCID50 or PBS in an equal volume of 50 µL on day 0, followed by an intravenous OTI T cell injection (1 × 10^6^) on day 1 for combination treatment or OTI monotherapy. Virus/PBS treatment was repeated on days 7 and 14. Tumors from both flanks were harvested on day 8, 15 and 22 after the first treatment. Single cell suspensions were generated from tumor tissues, and lymphocytes were isolated by gradient centrifugation. Tumor infiltrating lymphocytes (TILs) were then analyzed for CD8 surface expression (**A**) and OVA-specificity (**B**) via flow cytometry. Cell counts were normalized to tumor size measured before harvesting. Data are presented as mean + SD, and statistical significance was determined by student’s t-test. (**C**) Tumor size was correlated with OVA-specific T cell infiltration in a nonparametric Spearman correlation using GraphPad.

To analyze the kinetics of the treatment responses, mice were implanted with B16-OVA tumors on both flanks, and treatment was applied as in the previous experiment (Figure 5A). The mice were randomized for euthanasia on days 8, 15, and 22 for analysis of tumors, blood, and spleens. Later timepoints were chosen in this experiment to allow time for a sufficient induction of the adaptive immune response. Similar to the data gathered from the previous kinetics experiment (Figure 4), an increase of CD8+ tumor infiltrating lymphocytes was detected in the combination group by day 8 (Figure 6A). Here, the results were normalized to tumor size in order to account for variability of lymphocyte cell number isolated by gradient centrifugation. With a slight delay, this increase was also detected in VSV-NDV treated tumors by day 15. Although not significant, this trend, taken together with a similar trend observed for the infiltration of OVA-specific TILs normalized to tumor size (Figure 6B), indicates that adoptively transferred T cells preferentially infiltrate and expand in tumors that had a prior infection with VSV-NDV, thereby contributing to the enhanced therapeutic effect. This effect could not be observed in uninjected tumors. Due to the substantial reduction in tumor size of the injected tumors in the combination group by day 22, the normalized OVA-specific population observed here is especially impressive. A negative correlation between the enrichment of OVA-specific cytotoxic T cells and tumor size of the injected tumors (Figure 6C) indicates that the T cells likely play an important role in tumor clearance in this setting. This correlation could not be confirmed in the uninjected tumors, pointing to the importance of VSV-NDV pre-infection of the tumor in mediating the therapeutic response of ACT.

## 4. Discussion

With the recent approval of CAR T cell therapies targeting CD19 [21], as well as numerous other T cell therapies currently under clinical investigation, the concept of adoptive T cell therapy for cancer is at the forefront of the immune-oncology field. However, despite the promising responses to these therapies in specific subsets of patients, a weakness of this approach is that it relies on the identification of suitable tumor-associated antigens (TAAs) and neoantigens to target. Due to intratumoral heterogeneity and the process of cancer immune-editing, strategies that target a single antigen can result in the selection for tumor cells which do not express the targeted antigen, leading to escape variants causing relapse [22]. Furthermore, since each tumor has its own distinct gene signature, targeted therapies often require expensive and time-consuming molecular screening of tumor biopsies and subsequent production of personalized treatments. Therefore, the concept of combining ACT approaches with oncolytic virus therapy has emerged as a rational strategy to, not only broaden the scope of the response by using the virus to kill cells that are not targeted by the ACT, but also to potentially enhance the infiltration and expansion of adoptively transferred cells through pro-inflammatory signaling mediated by the virus infection. This approach therefore has the potential to drive multiple mechanisms leading to synergistic tumor debulking and long-term systemic protection against the tumor.

In the study reported here, we combined a highly immune-stimulatory oncolytic virus candidate with adoptive TCR T cell therapy in a model mouse system for melanoma, in order to demonstrate proof-of-concept and a preliminary mechanism to support the further development of the approach. Our results emphasize that the combination of oncolytic virus and antigen-specific T cells can work together to beneficially modulate the tumor microenvironment, including the induction of MHC-I expression on tumor cells (Figure 2F,G), which is essential for an efficient elimination by antigen-specific T cells [23]. Synergy was demonstrated, not only through the complementary mechanisms of viral-mediated oncolysis and T cell effector functions, but also through the important finding that T cells seem to be specifically recruited to areas of VSV-NDV infection (Figure 2H), potentially enhancing the contribution of the adoptively transferred T cells in the combination therapy, as shown previously [24,25]. Although the mechanism remains unclear, this finding is supported by the observation of an influx of CD8^+^ tumor infiltrating lymphocytes in response to combination therapy in vivo (Figure 4A and Figure 6A). OVA-specific infiltration was significantly increased compared to OTI monotherapy in both the injected and uninjected tumor at early timepoints (Figure 4A, upper panel). An improved infiltration of adoptively transferred T cells compared to VSV-NDV monotherapy could not be confirmed after a single virus injection, but data suggest an infiltration of adoptively transferred T cells and an accelerated expansion of OVA-specific T cells after multiple virus injections compared to VSV-NDV monotherapy. This could not be observed in uninjected tumors indicating a required threshold of infection to attract the adoptively transferred population into the tumor. Research on transgene-expressing oncolytic viruses in combination therapy suggests that this effect could be further improved by engineering VSV-NDV to express cytokines, chemokines or other T cell engagers, such as BiTEs. A TNFα- and IL-12-expressing adenovirus has been shown to improve ACT in different combination approaches [26,27,28]. Similarly, a vaccinia virus expressing the chemokine CXCL11 or IL15Rα enhanced the anti-tumor activity of CAR T cells in solid tumors [29,30].

The finding of virus-specific T cells in the uninjected tumor (Figure 4A, upper panel) was unexpected and warrants further investigation. Since we failed to detect infectious virus in contralateral uninjected tumors in previous experiments (data not shown), we suspect that the presence of virus-specific T cells in the uninjected tumor is a reflection of the systemic accumulation of these cells at the investigated time-point, rather than being an indication of a specific intratumoral infiltration in response to virus spread to the uninjected tumor. This hypothesis will be explored in follow-up studies.

The in vivo studies shown here indicate that adoptively transferred tumor-specific TCR T cells lead to only negligible tumor responses, when administered as a monotherapy (Figure 3C and Figure 5B). This was likely due to the relatively low number of T cells injected, as well as challenges of effective T cell engraftment, both of which are relevant limitations that are faced in clinical application. Lymphodepletion plays a key role in improving engraftment of transferred T cells. Cole et al. showed that the anti-tumor effects of VSV combined with OTI T cells were further improved in lymphopenic hosts [31]. In contrast, not only was the hybrid VSV-NDV virus extremely effective in delaying tumor growth in the injected tumor, but it also led to impressive abscopal effects in contralateral uninjected tumors, when injected frequently over the course of one week. By reducing the injection frequency to a more clinically applicable dosing scheme, we could then appreciate the power of the combination therapy, which led to a survival prolongation and significant delay in tumor growth of injected and distant tumors, with more than 30% of treated mice achieving long-term remission.

## 5. Conclusions

Our results demonstrate the robustness of the combination treatment approach when applied in a clinically relevant dosing scheme and without prior lymphodepletion. This is promising, in that this treatment would be available to a broader patient population, without an aggressive myeloablative radiation protocol [32]. Furthermore, the effectiveness of combination therapy in conditions where monotherapy showed no effect, could suggest a better chance of treatment response, despite unknown variables of virus and T cell kinetics in the patient.

The first clinical trials combining oncolytic adenovirus and ACT are on the way to demonstrate the clinical benefit of this approach and its relevance in patient treatment (NCT03740256, NCT04217473). Despite promising preclinical data [26], the clinical outcome will depend on numerous factors, including the immunogenicity of the virus and its potency in T cell recruitment, as well as the efficiency of the adoptive T cell therapy. The full potential of ACT and the immune response induced by the combination therapy may be limited by prevailing suppressive signaling pathways, immune checkpoints or secondary escape mechanisms. This calls for a third partner in the combination approach, i.e., immune checkpoint inhibitors. On their own, checkpoint inhibitors already stand out as extremely promising treatment options for several tumor indications [33], but their systemic administration is still limited by the onset of severe adverse events [34], and response rates are dependent on many pre-existing features within the tumor, such as a high mutational load [35,36]. Oncolytic viruses can upregulate the local expression of immune checkpoints at the tumor site and create changes in the tumor microenvironment to sensitize the tumor to immune checkpoint blockade [37]. A triple combination approach, using adenovirus expressing PD-L1 antibody with CAR T cells, demonstrated therapeutic improvements in preclinical experiments [38,39,40]. Of course, the complexity and high costs of combination treatment approaches in their transfer to the clinic is greatly increased by the addition of more therapeutic agents, but it seems that a curative outcome may depend on targeting multiple mechanisms [22]. We predict that as we rapidly gain a new understanding of the biology of tumor pathogenesis, new rationally designed combination approaches will emerge. Due to the multi-mechanistic nature of oncolytic VSV-NDV, as well as its ability to optimally synergize with other immunotherapeutic agents, as shown here, it represents an attractive candidate for combinatorial approaches in immune-oncology.

## Data Availability

The data are available within this article and supplemental material. Original raw data are available upon request to the corresponding author.

## Supplementary Materials

The following are available online at https://www.mdpi.com/2072-6694/13/5/1044/s1, Figure S1: VSV-NDV replication kinetics and cytotoxicity in B16-F10 cells. Figure S2: Graphical representation for tumor cell staining of PD-L1 after co-culture. Figure S3: Experimental set-up of kinetics experiment. Figure S4: Analysis of blood cytokines in response to VSV-NDV and/or OTI T cell therapy. Figure S5: Analysis of individual tumor growth.
