# Supplementary material for: Adoptive T Cell Therapy Is Complemented by Oncolytic Virotherapy with Fusogenic VSV-NDV in Combination Treatment of Murine Melanoma
Source: Cancers (Basel). 2021 Mar 2;13(5):1044. doi: 10.3390/cancers13051044 (PMC7958625; doi:10.3390/cancers13051044)
Supplement: Supplementary file 1 [file cancers-13-01044-s001.zip › Supplementary files/Supplement Figure Legend.docx]

**Sup. Fig. 1: VSV-NDV replication kinetics and cytotoxicity in B16-F10 cells.**  B16-F10 cells were seeded and infected with VSV-NDV at different MOIs. Left panel: Virus titers were determined via a TCID50 assay from tissue culture supernatants collected at indicated times after infection. Right panel: Cytotoxicity of VSV-NDV infection was determined via an LDH detection assay. Results were normalized to a maximum release control. Means of experiments performed in triplicate + SEM are shown.

**Sup. Fig. 2: Graphical representation for tumor cell staining of PD-L1 after co-culture.**Co-culture experiments were performed by seeding B16 cells, pre-infecting them with VSV-NDV (VN) at MOI 0.01 for 16 h before adding OTI T cells (OTI) in a 1:1 effector-to-target ratio. Tumor cells were harvested 6 and 24 h after OTI addition and stained for flow cytometric analysis of PD-L1 expression. Mean fluorescence intensity (MFI) was determined from the same samples. Representative dot plots and histograms are shown for the indicated conditions.

**Sup. Fig. 3: Experimental set-up of kinetics experiment.** C57BL6/J mice were implanted with 2.4x10^5^ B16-OVA cells injected subcutaneously into both flanks. One week later, mice were randomly distributed into treatment groups (n = 5-6) and injected intratumorally (right tumor) with VSV-NDV at a dose of 10^7^ TCID50 or PBS in an equal volume of 50 μl on day 0, followed by an intravenous OTI T cell injection (5.5x10^6^) on day 1 for combination treatment or OTI monotherapy. Mice were then randomized for sacrifice on day 2, 5 or 8, and tumors, blood and spleens were harvested for further analysis.

**Sup. Fig. 4**: **Analysis of blood cytokines in response to VSV-NDV and/or OTI T cell therapy.** The cytokine levels were analyzed from plasma prepared from subcutaneous B16-OVA tumor-bearing mice on day 2, 5 and 8 after treatment initiation. A bead-based cytokine array (BioLegend) was performed according to the manufacturer’s protocol and analyzed via flow cytometry. The data showing plasma concentrations of IL-1α, IFNγ, MCP-1 and IL-27 over time after the indicated treatment are shown as means + standard error of the mean.

**Sup. Fig. 5: Analysis of individual tumor growth.**  C57BL6/J mice were implanted with 2.4x10^5^ (injected tumor) and 1.2x10^5^ (uninjected tumor) B16-OVA cells subcutaneously and randomized into treatment groups (n = 5-6) one week later. They were injected intratumorally with VSV-NDV at a dose of 10^7^ TCID50 or PBS in an equal volume of 50 μl on day 0, followed by an intravenous OTI T cell injection (5.5x10^6^) on day 1 for combination treatment or OTI monotherapy. Intratumoral virus or PBS injections were repeated on day 7 and 14. Tumor growth was monitored by caliper measurements of tumor width and length. The volume was calculated according to the modified ellipsoid formula: Tumor volume = 1/2(length x width^2^). Tumor volumes for individual tumors were plotted for injected (gray) and uninjected (black) tumors, according to the indicated treatment group.
