# Supplementary figures and images for: Adoptive T Cell Therapy Is Complemented by Oncolytic Virotherapy with Fusogenic VSV-NDV in Combination Treatment of Murine Melanoma
Source: Cancers (Basel). 2021 Mar 2;13(5):1044. doi: 10.3390/cancers13051044 (PMC7958625; doi:10.3390/cancers13051044)

**Sup. Fig. 5**

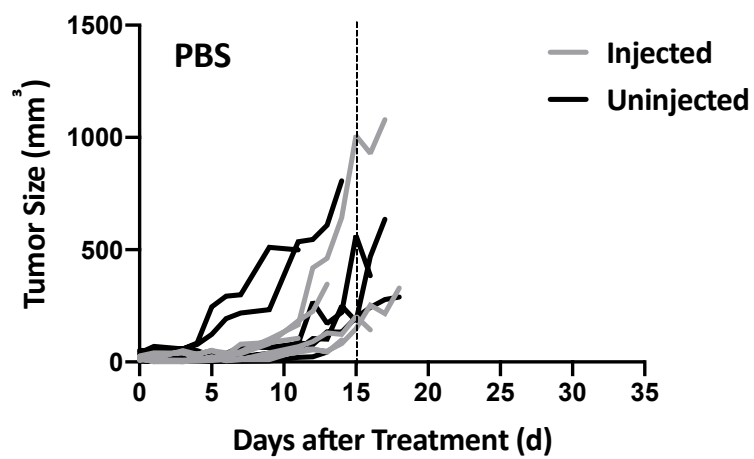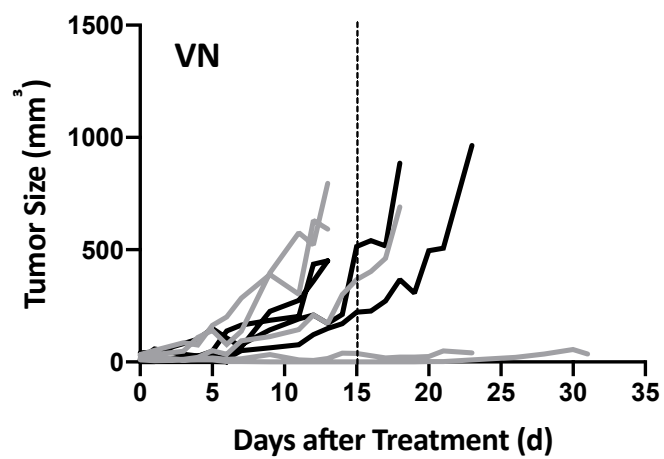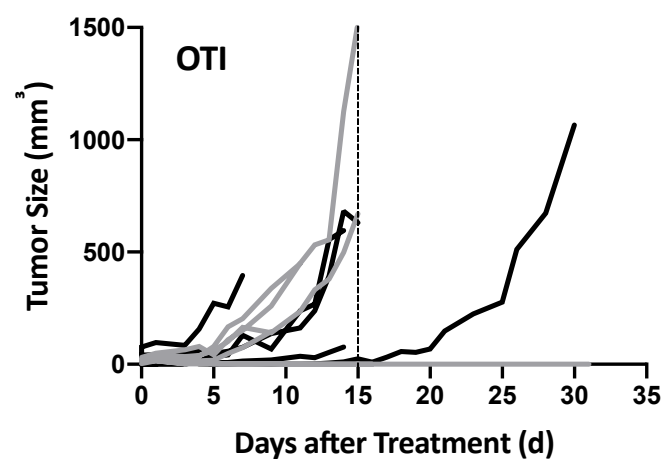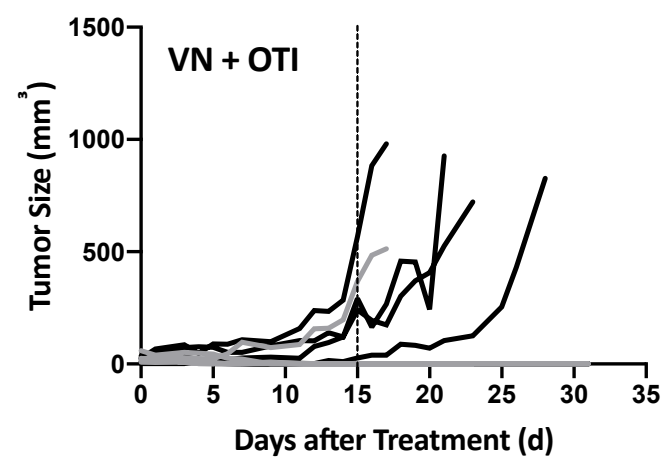

Supplement: Supplementary file 1 [file cancers-13-01044-s001.zip › Supplementary files/Sup_Figure 5.pdf]

Sup. Fig. 4

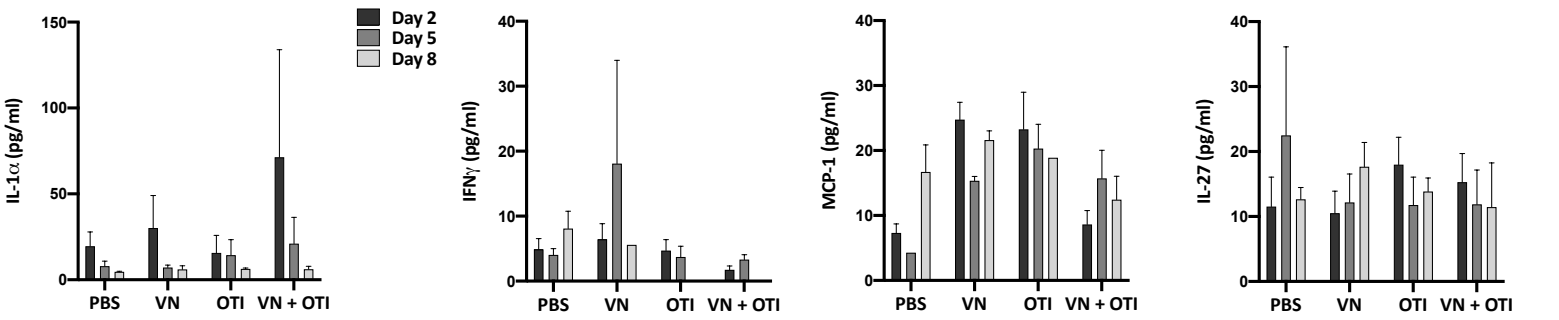

Supplement: Supplementary file 1 [file cancers-13-01044-s001.zip › Supplementary files/Sup_Figure 4.pdf]

# Sup. Fig. 3

Tumor Implantation  
B16-OVA (s.c.), both flanks  
7 days prior

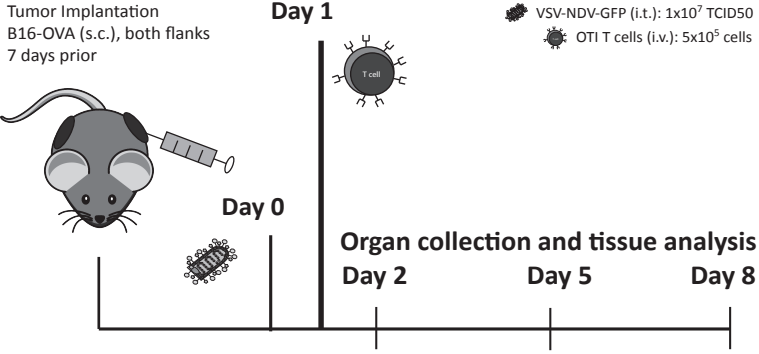

Supplement: Supplementary file 1 [file cancers-13-01044-s001.zip › Supplementary files/Sup_Figure 3.pdf]

Sup. Fig. 2

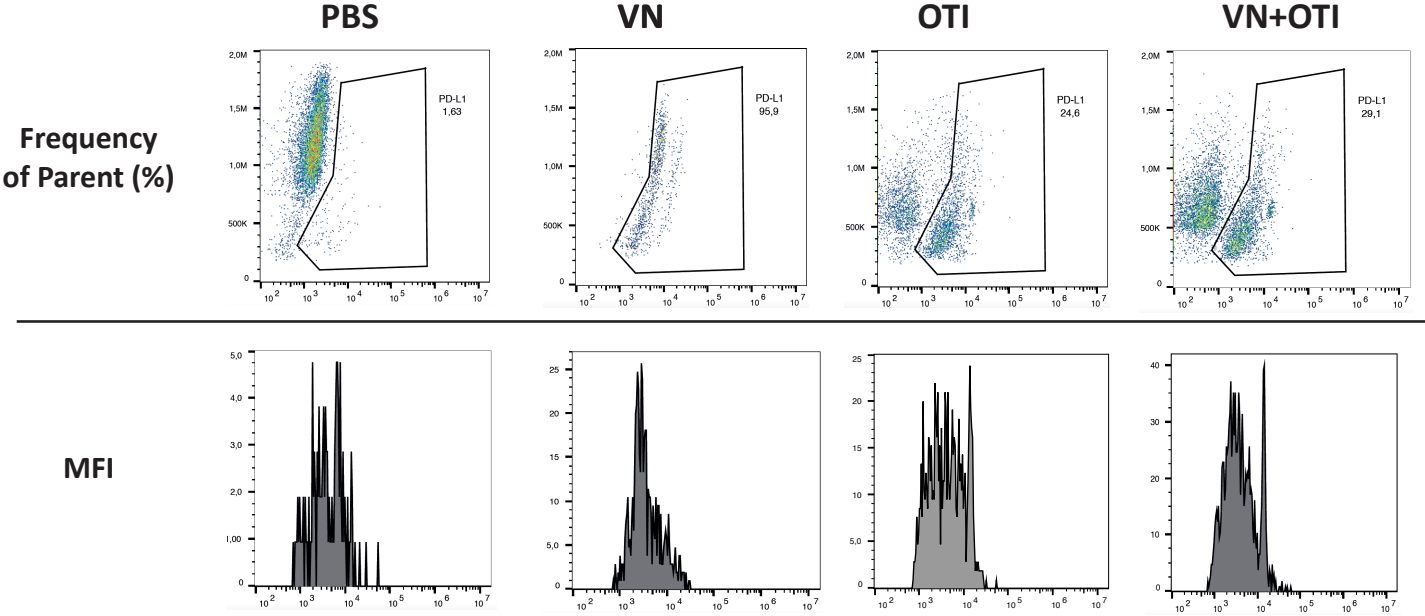

Supplement: Supplementary file 1 [file cancers-13-01044-s001.zip › Supplementary files/Sup_Figure 2.pdf]

Sup. Fig. 1

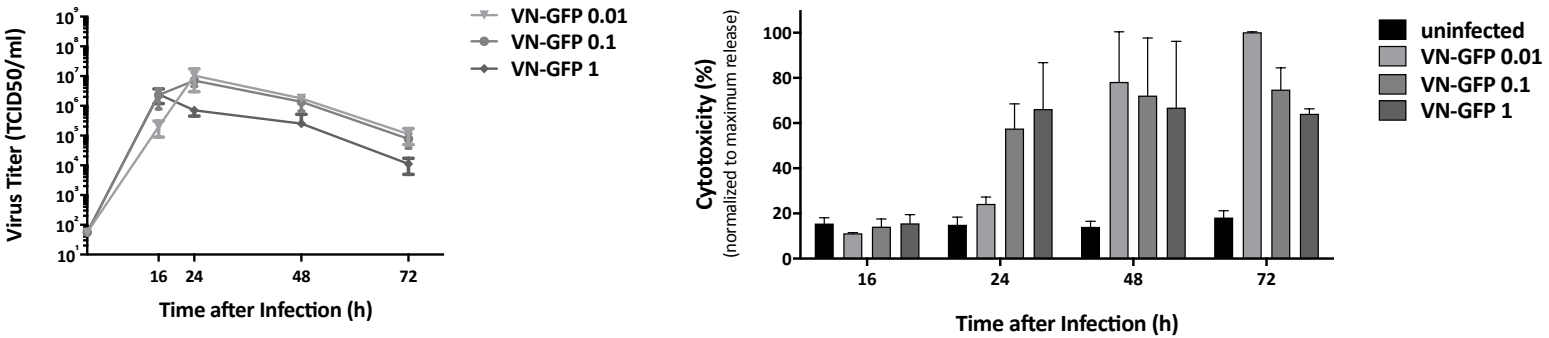

Supplement: Supplementary file 1 [file cancers-13-01044-s001.zip › Supplementary files/Sup_Figure 1.pdf]
